# Prediction of Major Adverse Cardiovascular Events by Triglyceride Glucose Index in Predominantly Male Patients with Rheumatoid Arthritis

**DOI:** 10.31083/j.rcm2501028

**Published:** 2024-01-15

**Authors:** Jiawei Zhang, Qiqi Hou, Quanle Han, Xu Peng, Hongxia Cao, Shouling Wu, Kangbo Li

**Affiliations:** ^1^Department of Rehabilitation Medicine, Kailuan Tangjiazhuang Hospital, 063000 Tangshan, Hebei, China; ^2^Hebei Medical University, 050017 Shijiazhuang, Hebei, China; ^3^Department of Cardiology, Tangshan Gongren Hospital, 063000 Tangshan, Hebei, China; ^4^Charité – Universitätsmedizin Berlin, Corporate Member of Freie Universität Berlin and Humboldt-Universität zu Berlin, 10117 Berlin, Germany; ^5^Catheterization Unit, Tangshan Gongren Hospital, 063000 Tangshan, Hebei, China; ^6^Department of Cardiology, Kailuan General Hospital, 063000 Tangshan, Hebei, China; ^7^School of Clinical Medicine, North China University of Science and Technology, 063000 Tangshan, Hebei, China

**Keywords:** rheumatoid arthritis, triglyceride glucose index, cardiovascular disease, myocardial infarction, stroke

## Abstract

**Background:**

Rheumatoid arthritis (RA) is a systemic and chronic autoimmune disease that is characterized by persistent joint inflammation. RA patients experience a considerably increased risk of cardiovascular-related morbidity and mortality. The current study investigated the association between triglyceride glucose (TyG) index and major adverse cardiovascular events (MACEs) in a predominantly male cohort of RA patients.

**Methods:**

A total of 1613 RA patients (81.53% male) were selected from the Kailuan study. The TyG index was calculated as the logarithmic product of fasting blood triglyceride and fasting blood glucose divided by two. MACEs were defined as the composite of non-fatal myocardial infarctions and non-fatal strokes. Cox proportional hazards analysis was performed to study the association between the TyG index and MACEs.

**Results:**

A total of 59 MACEs occurred during the median follow-up time of 5.32 years. Following adjustment for age and gender, analysis by multivariable Cox proportional hazards (model 1) showed that an elevated TyG index was associated with an increased risk of MACEs (quartile 2, hazard ratio (HR): 2.741, 95% confidence interval (CI): 1.220–6.157, *p* = 0.015; quartile 4, HR: 2.521, 95% CI: 1.074–5.917, *p* = 0.034). After adjustment for other variables, Cox proportional hazards analysis (model 2) showed that an elevated TyG index was independently associated with an increased risk of MACEs (quartile 2, HR: 2.348, 95% CI: 1.009–5.465, *p* = 0.048). In addition, subgroup analysis showed a higher TyG index was significantly linked to an increased risk of MACEs in patients aged more than 65 years (quartile 2, HR: 6.048, 95% CI: 1.311–27.908, *p* = 0.021; quartile 4, HR: 12.074, 95% CI: 1.438–101.358, *p* = 0.022).

**Conclusions:**

The TyG index was associated with an increased risk of MACEs in a predominantly male cohort of RA patients. This index may be helpful for the prediction of MACEs in male patients with RA.

**Clinical Trial Registration:**

Registration number in the Chinese clinical trial registry: ChiCTR-TNRC-11001489.

## 1. Introduction

Rheumatoid arthritis (RA) is a chronic, inflammatory, and disabling disease that affects about 18 million people worldwide [1]. The major consequence of RA is work disability [2], with a rate being relatively high at 20% to 35% after 5 years of the disease course [3, 4, 5]. Furthermore, RA patients have a 1.5-fold higher mortality rate than the general population [6]. The most frequently reported cause of death in RA patients is cardiovascular disease (CVD) [7, 8]. Indeed, RA is closely associated with a substantially increased risk of CVD, largely due to the presence of atherosclerosis (AS) [9], which is the thickening or hardening of the arteries due to the plaque accumulation in the inner arterial lining. In addition to traditional cardiovascular risk factors, chronic inflammation may also be regarded as an independent risk factor for AS in RA patients [10].

Recently, a large body of evidence supports the notion that insulin resistance (IR) is critical in both RA and CVD [11, 12]. Indeed, the triglyceride glucose (TyG) index may be considered a surrogate marker for IR [13]. The systemic inflammation associated with RA contributes to the development of abnormal lipid and glucose metabolisms [14, 15]. Recent studies have also shown that the TyG index is a strong and useful predictor of cardiovascular outcomes in various disease settings [16, 17]. However, investigations into potential associations between the TyG index and cardiovascular outcomes in RA patients are still lacking, especially in males. Hence, the current study aimed to investigate whether the TyG index could be used to predict major adverse cardiovascular events (MACEs) in a predominantly male cohort of RA patients.

## 2. Patients and Methods

### 2.1 Patient Cohort

**Fig. 1.**
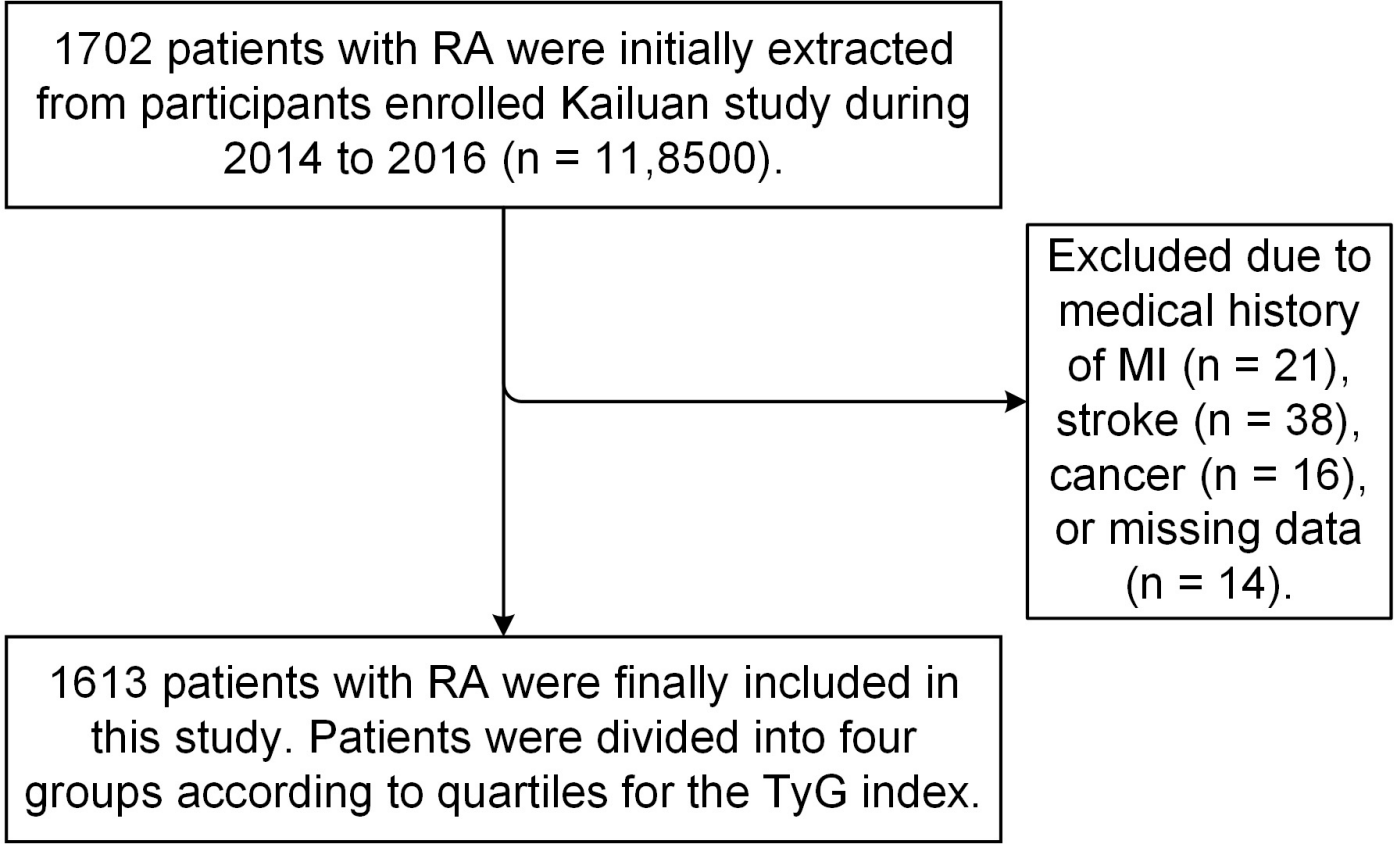
**Flowchart of this study**. RA, rheumatoid arthritis; MI, myocardial infarction; TyG index, triglyceride glucose index.

A total of 1702 patients diagnosed with RA during 2014–2016 were initially extracted from 118,500 participants in the Kailuan study database. Of these, 89 patients with histories of myocardial infarction (MI) (n = 21), stroke (n = 38), and cancer (n = 16), or with missing data (n = 14) were excluded, leaving 1613 RA patients that were included in the current study (Fig. 1).

### 2.2 Triglyceride Glucose Index Trajectory

Fasting blood tests were performed for all patients, with all samples being processed in the central laboratory of the Kailuan General Hospital. The TyG index was calculated: ln (fasting triglycerides (mg/dL) × fasting glucose (mg/dL)/2). All patients were categorized into four groups based on quartiles for the TyG index.

### 2.3 Follow-Up and Study Endpoint

All patients were followed until December 2020, with a median of 5.3 years. The study endpoint was defined as the occurrence of a MACE, including MI, ischemic stroke, and hemorrhagic stroke.

### 2.4 Statistical Analysis

Categorical variables were presented as proportions, and continuous variables as mean ± standard deviation or median and interquartile range. Kaplan–Meier analysis was used to evaluate the cumulative incidence of the study endpoint over time. Two Cox proportional hazards models were used to investigate associations between variables with MACEs. Statistical significance was set at *p* < 0.05.

## 3. Results

### 3.1 Patient Baseline Characteristics

Table 1 shows the baseline characteristics for the 1613 RA patients included in this study. The mean age of the patients was 55.41 ± 12.43 years, while 1315 (81.53%) were male. The mean TyG index was 8.81 ± 0.74. Patients were divided into four groups based on the baseline TyG index quartiles: quartile 1 (6.40 ≤ TyG < 8.28), quartile 2 (8.28 ≤ TyG < 8.73), quartile 3 (8.73 ≤ TyG < 9.26), and quartile 4 (9.26 ≤ TyG ≤ 11.56).

**Table 1.** **Baseline characteristics of patients according to quartiles of TyG index**.

Variables	Total (n = 1613)	Quartile 1 (n = 403)	Quartile 2 (n = 403)	Quartile 3 (n = 404)	Quartile 4 (n = 403)	*p* value
Age, years	55.41 ± 12.43	54.17 ± 13.38	55.59 ± 12.83	56.54 ± 11.67	55.33 ± 11.70	0.125
Male, n (%)	1315 (81.53)	336 (83.37)	323 (80.15)	321 (79.46)	335 (83.13)	0.516
BMI, ${\mathrm{kg/m}}^{2}$	25.29 ± 3.29	23.93 ± 3.11	24.86 ± 3.01	26.06 ± 3.35	26.31 ± 3.10	<0.0001
SBP, mmHg	137.40 ± 19.66	130.40 ± 17.95	136.36 ± 19.96	140.58 ± 19.79	142.23 ± 18.79	<0.0001
DBP, mmHg	81.80 ± 11.01	78.73 ± 10.36	80.93 ± 10.38	82.95 ± 11.49	84.60 ± 10.90	<0.0001
FBG, mmol/L	6.00 ± 1.89	5.20 ± 0.72	5.59 ± 0.93	6.13 ± 1.52	7.09 ± 2.94	<0.0001
TC, mmol/L	5.01 ± 1.36	4.80 ± 0.86	5.12 ± 1.02	5.29 ± 0.97	4.82 ± 2.13	<0.0001
TG, mmol/L	1.36 (0.90–2.09)	0.72 (0.60–0.85)	1.13 (1.00–1.29)	1.65 (1.45–1.89)	3.37 (2.50–4.56)	<0.0001
HDL-C, mmol/L	1.37 ± 0.35	1.49 ± 0.35	1.38 ± 0.34	1.31 ± 0.30	1.30 ± 0.39	<0.0001
LDL-C, mmol/L	2.91 ± 0.77	2.71 ± 0.67	2.93 ± 0.77	3.04 ± 0.73	2.97 ± 0.86	<0.0001
hs-CRP, mg/L	1.25 (0.40–2.70)	0.91 (0.34–2.25)	1.24 (0.40–2.30)	1.45 (0.63–2.85)	1.30 (0.40–2.90)	0.011
TyG index	8.81 ± 0.74	7.95 ± 0.28	8.51 ± 0.13	8.96 ± 0.15	9.81 ± 0.45	<0.0001
Hypertension, n (%)	1015 (62.93)	194 (48.14)	237 (58.81)	284 (70.30)	300 (74.44)	<0.0001
Diabetes, n (%)	294 (18.23)	32 (7.94)	42 (10.42)	84 (20.79)	136 (33.75)	<0.0001
Snoring, n (%)	615 (38.13)	128 (31.76)	162 (40.20)	173 (42.82)	152 (37.72)	0.022
Smoking, n (%)	753 (46.68)	187 (46.40)	178 (44.17)	182 (45.05)	206 (51.12)	0.325
Drinking, n (%)	482 (29.88)	104 (25.81)	129 (32.01)	113 (27.97)	136 (33.75)	0.106

TyG index, triglyceride glucose index; BMI, body mass index; SBP, systolic blood pressure; DBP, diastolic blood pressure; FBG, fasting blood glucose; TC, total cholesterol; TG, triglyceride; HDL-C, high-density lipoprotein cholesterol; LDL-C, low-density lipoprotein cholesterol; hsCRP, high-sensitivity C-reactive protein.

Patients in quartile 4 were more likely to have higher body mass index, systolic blood pressure, and diastolic blood pressure, alongside higher levels of fasting blood glucose and triglyceride. Quartile 4 patients also had higher incidences of hypertension and diabetes.

### 3.2 Incidence of Major Adverse Cardiovascular Events

**Fig. 2.**
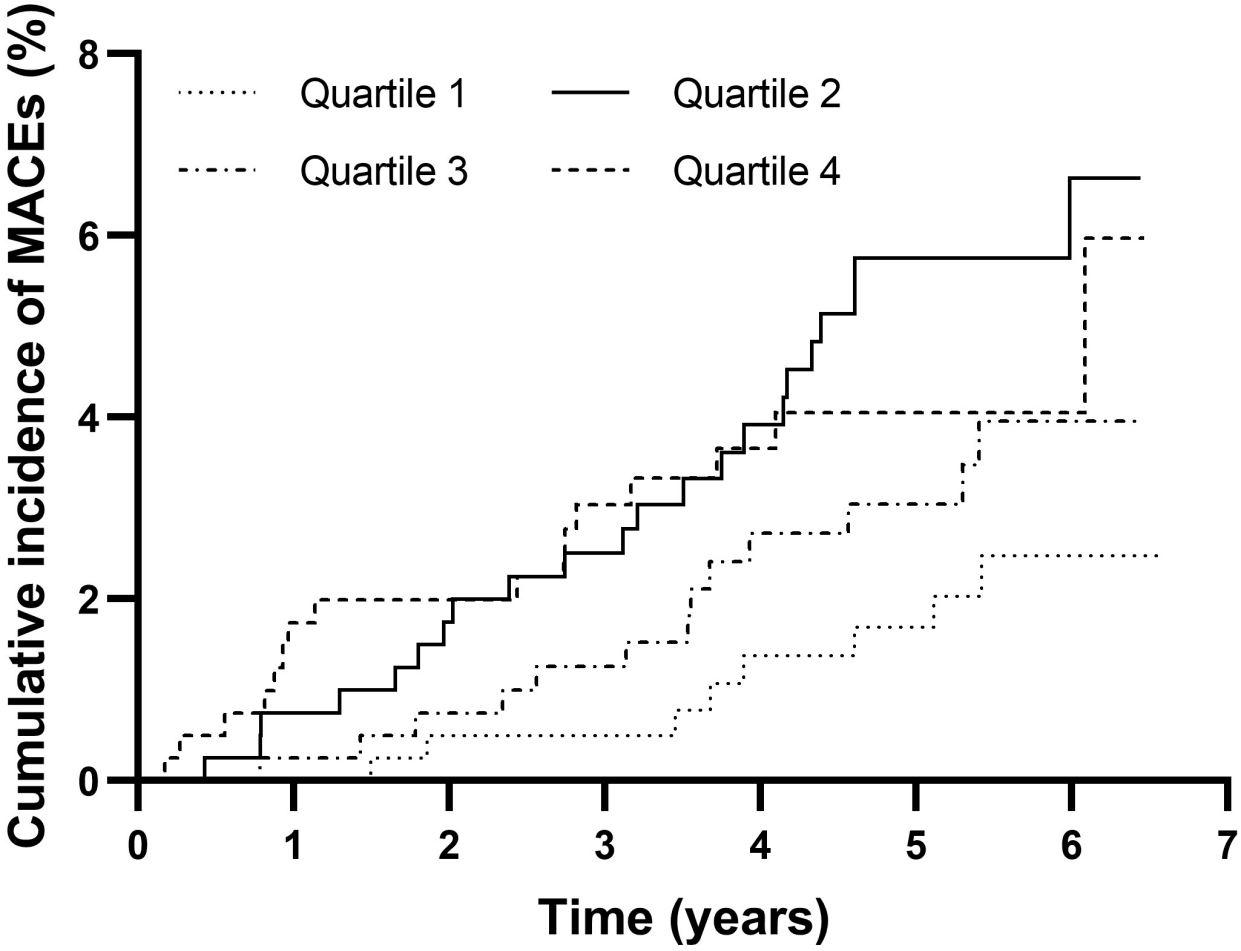
**Cumulative incidence of MACEs in the TyG index quartile groups**. MACEs, major adverse cardiovascular events; TyG index, triglyceride glucose index.

A total of 59 MACEs were documented during the median follow-up period of 5.32 years. A total of 8 MACEs occurred in quartile 1, 22 occurred in quartile 2, 13 in quartile 3, and 16 in quartile 4. As shown in Fig. 2, patients in quartile 2 had the highest cumulative incidence of MACEs, although a significant difference was not reached (quartile 1: 2.48%, quartile 2: 6.63%, quartile 3: 3.96%, quartile 4: 4.05%, *p* = 0.060).

As shown in Table 2, multivariate Cox proportional hazards regression analysis (model 1) revealed that a higher TyG index was significantly associated with an increased risk of MACEs (quartile 2, hazard ratio (HR): 2.741, 95% confidence interval (CI): 1.220–6.157, *p* = 0.015; quartile 4, HR: 2.521, 95% CI: 1.074–5.917, *p* = 0.034). Quartile 3 in the TyG index was also positively associated with an increased risk of MACEs, although this did not reach statistical significance (HR: 1.645, 95% CI: 0.681–3.972, *p* = 0.268). Age was significantly associated with MACEs (HR: 1.066, 95% CI: 1.043–1.090, *p* < 0.0001) (Table 3). After adjusting for other confounding cardiovascular risk factors (model 2), the TyG index remained significantly associated with MACEs in patients in quartile 2 (HR: 2.348, 95% CI: 1.009–5.465, *p* = 0.048), although not in patients in quartile 3 (HR: 1.330, 95% CI: 0.489–3.617, *p* = 0.576) or quartile 4 (HR: 2.614, 95% CI: 0.694–9.843, *p* = 0.156) (Table 2). A significant association between age and MACEs was again shown in model 2 (HR: 1.073, 95% CI: 1.043–1.104, *p* < 0.0001).

**Table 2.** **Adjusted hazard ratios for MACEs in TyG index quartile groups**.

		β	SE	χ ^2^	*p* value	HR	95% CI
Adjusted model 1							
	Quartile 2	1.008	0.413	5.961	0.015	2.741	1.220–6.157
	Quartile 3	0.498	0.450	1.226	0.268	1.645	0.681–3.972
	Quartile 4	0.925	0.435	4.515	0.034	2.521	1.074–5.917
	Age	0.064	0.011	32.809	<0.0001	1.066	1.043–1.090
	Gender (male)	0.431	0.363	1.414	0.234	1.539	0.756–3.132
Adjusted model 2							
	Quartile 2	0.854	0.431	3.922	0.048	2.348	1.009–5.465
	Quartile 3	0.285	0.510	0.313	0.576	1.330	0.489–3.617
	Quartile 4	0.961	0.677	2.017	0.156	2.614	0.694–9.843
	Age	0.070	0.015	23.191	<0.0001	1.073	1.043–1.104
	Gender (male)	0.102	0.417	0.060	0.807	1.107	0.489–2.506
	BMI	0.032	0.044	0.527	0.468	1.033	0.947–1.126
	SBP	0.000	0.008	0.000	0.991	1.000	0.984–1.016
	DBP	0.012	0.015	0.660	0.417	1.012	0.983–1.041
	FBG	0.118	0.075	2.454	0.117	1.126	0.971–1.305
	TC	0.092	0.128	0.524	0.469	1.097	0.854–1.408
	TG	–0.201	0.176	1.306	0.253	0.818	0.579–1.155
	HDL-C	–0.456	0.470	0.942	0.332	0.634	0.252–1.592
	LDL-C	0.058	0.218	0.072	0.789	1.060	0.692–1.624
	hs-CRP	0.012	0.019	0.401	0.527	1.012	0.975–1.050
	Hypertension	0.328	0.373	0.775	0.379	1.388	0.669–2.883
	Diabetes	–0.413	0.408	1.023	0.312	0.662	0.298–1.472
	Snoring	0.128	0.274	0.218	0.640	1.137	0.664–1.947
	Smoking	0.378	0.291	1.693	0.193	1.459	0.826–2.579
	Drinking	0.442	0.306	2.094	0.148	1.557	0.855–2.834

Note: Model 1 was adjusted for age and gender (male). Model 2 was adjusted for age and gender (male), body mass index, systolic blood pressure, diastolic blood pressure, fasting blood glucose, total cholesterol, triglyceride, high-density lipoprotein cholesterol, low-density lipoprotein cholesterol, high-sensitivity C-reactive protein, hypertension, diabetes, snoring, smoking, and drinking. MACEs, major adverse cardiovascular events; TyG index, triglyceride glucose index; SE, standard error; HR; hazard ratio; CI, confidence interval; BMI, body mass index; SBP, systolic blood pressure; DBP, diastolic blood pressure; FBG, fasting blood glucose; TC, total cholesterol; TG, triglyceride; HDL-C, high-density lipoprotein cholesterol; LDL-C, low-density lipoprotein cholesterol; hsCRP, high-sensitivity C-reactive protein.

**Table 3.** **Major adverse cardiovascular events in age and TyG index quartile subgroups**.

Subgroup			Total (event)	*p* value	HR	95% CI	*p* for interaction
Age							0.227
	≥65 years						
		Quartile 1	91 (6)	-	-	-	
		Quartile 2	96 (9)	0.021	6.048	1.311–27.908	
		Quartile 3	89 (4)	0.076	4.530	0.853–24.066	
		Quartile 4	86 (6)	0.022	12.074	1.438–101.358	
	<65 years						
		Quartile 1	312 (2)	-	-	-	
		Quartile 2	307 (13)	0.848	0.891	0.273–2.911	
		Quartile 3	315 (9)	0.316	0.420	0.077–2.288	
		Quartile 4	317 (10)	0.763	0.672	0.051–8.874	

TyG index, triglyceride glucose index; HR; hazard ratio; CI, confidence interval.

As shown in Table 3, further subgroup analysis showed that a higher TyG index was significantly associated with the risk of MACEs in elderly patients (≥65 years) (quartile 2, HR: 6.048, 95% CI: 1.311–27.908, *p* = 0.021; quartile 4, HR: 12.074, 95% CI: 1.438–101.358, *p* = 0.022). No significant interactions were found between the subgroups.

## 4. Discussion

Numerous studies have shown that RA patients are at greater risk of CVD compared with the general population [18, 19]. The excess risk is due to RA-specific risk-factor profiles, such as systemic inflammation, rather than traditional CVD risk factors [20]. Furthermore, RA can be regarded as an independent risk factor for CVD [21]. Together with risk-based CVD management, the use of anti-rheumatic medication is fundamental procedure for reducing the incidence of CVD [22]. Still, it has been found that RA is associated with an increased risk of CVD [23]. Solomon *et al*. [24] reported that female RA patients had a significantly higher risk of MI compared to those without RA. Specifically, for women with a medical history of RA of at least 10 years, the adjusted relative risk (RR) was 3.10, and for those with a medical history of RA less than 10 years, the RR was 1.16 [24]. Fischer *et al*. [25] also reported an increased risk of acute MI in patients with RA, with an adjusted odds ratio (OR) of 1.47. Södergren *et al*. [26] found that RA patients had higher morbidities from acute MI than the general population, with all-cause mortality showing an HR of 1.67. Holmqvist *et al*. [27] reported a rapid increase in the risk of MI within 1–4 years after RA diagnosis.

Similarly, both the morbidity and mortality from stroke were found to be higher in RA patients than in the general population [28]. Trömmer *et al*. [29] reported that RA was associated with both stroke (HR = 1.42) and transient ischemic attack (TIA) (HR = 1.69). Furthermore, patients aged 18–40 years showed the highest risk group for stroke (HR = 3.45) [29]. However, Holmqvist *et al*. [27, 30] reported that the progress of ischemic stroke in RA patients was slower than for ischemic heart disease. Moreover, Tiosano* et al*. [31] reported that RA was independently associated with stroke (OR = 1.18), particularly among young adults aged less than 65 years (OR = 1.79). Meissner *et al*. [32] reported that adverse events, particularly serious infections, and inadequate CVD treatment increased the risk of stroke in RA patients.

Insulin resistance can be defined as a pathological condition in which normal insulin concentrations induce subnormal biological effects [33]. Thus, the TyG index is more cost-effective and readily available for the direct measurement of IR compared to the gold standard, which is the hyperinsulinemic-euglycemic clamp. Therefore, the TyG index can be considered a surrogate marker for IR [34]. Furthermore, an increasing body of evidence suggests that the TyG index can be used to predict MACEs in different disease settings [35, 36, 37, 38].

As far as we are aware, this is the first study to investigate the TyG index in relation to MACEs in a predominantly male cohort of RA patients. We found that a higher TyG quartile (quartile 2: 8.28 ≤ TyG < 8.73) was associated with an increased risk of MACEs. However, a cut-off value for TyG could not be confirmed because the quartile 3 and quartile 4 groups did not show statistically significant associations with MACEs.

Since the Kailuan group is a company in the coal industry, considerably more males than females were employed, meaning that over 80% of the RA patients in this study were males. Our results are in contrast with the 3-fold higher incidence of RA found in women in the general population compared to men. Therefore, it is unclear whether the current findings are applicable to a more general RA population. On the one hand, CVD has long been regarded as a “male” disease [39], since males have an inherently greater risk for MACEs occurring than females. Research on gender differences in CVD indicates that males may not cope with stress as well as females, either physiologically, behaviorally, or emotionally, thereby increasing their risk of MACEs occurring. Stress-related factors can cause direct effects by influencing physiological parameters associated with the risk of CVD, as well as asserting indirect effects by increasing unhealthy behavioral and emotional responses associated with CVD (e.g., smoking, alcohol consumption, and depression) [40]. Alternatively, RA is known to affect about two to three times more women than men. Sex hormones play a critical role in regulating the immune response and are therefore strongly associated with the etiology of RA [41]. Presently, there has been a lack of research regarding cardiovascular risk factors for RA in men. Indeed, as far as we are aware, this is the first study to investigate the TyG index in relation to MACEs in a predominantly male cohort of RA patients. However, further research is needed to confirm whether the TyG index is a predictor of MACEs in RA patients.

## 5. Conclusions

In summary, our study found that the TyG index was associated with an increased risk of MACEs in a predominantly male cohort of RA patients. These results suggest that the TyG index is a potentially useful predictor of MACEs in predominantly male patients with RA. The pathological mechanism of IR and its effect on cardiovascular outcomes in RA patients warrants further investigation.

## Data Availability

The data that support the findings of this study are available from the corresponding author upon reasonable request.
